# Craniofacial and cardiac defects in *chd7* zebrafish mutants mimic CHARGE syndrome

**DOI:** 10.3389/fcell.2022.1030587

**Published:** 2022-12-07

**Authors:** Yuhan Sun, S. Ram Kumar, Chee Ern David Wong, Zhiyu Tian, Haipeng Bai, J. Gage Crump, Ruchi Bajpai, Ching Ling Lien

**Affiliations:** ^1^ Saban Research Institute and Heart Institute, Children’s Hospital Los Angeles, Los Angeles, CA, United States; ^2^ Center for Craniofacial Molecular Biology, Ostrow School of Dentistry, University of Southern California, Los Angeles, CA, United States; ^3^ Department of Surgery, Keck School of Medicine, University of Southern California, Los Angeles, CA, United States; ^4^ State Key Laboratory of Chemical Oncogenomics, Key Laboratory of Chemical Genomics, Peking University Shenzhen Graduate School, Shenzhen, China; ^5^ Eli and Edythe Broad CIRM Center for Regenerative Medicine and Stem Cell Research, Keck School of Medicine, University of Southern California, Los Angeles, CA, United States; ^6^ Department of Biochemistry and Molecular Medicine, Keck School of Medicine, University of Southern California, Los Angeles, CA, United States

**Keywords:** CHD7, CHARGE syndrome, craniofacial development, cardiovascular, aortic arch arteries, zebrafish

## Abstract

Congenital heart defects occur in almost 80% of patients with CHARGE syndrome, a sporadically occurring disease causing craniofacial and other abnormalities due to mutations in the *CHD7* gene. Animal models have been generated to mimic CHARGE syndrome; however, heart defects are not extensively described in zebrafish disease models of CHARGE using morpholino injections or genetic mutants. Here, we describe the co-occurrence of craniofacial abnormalities and heart defects in zebrafish *chd7* mutants. These mutant phenotypes are enhanced in the maternal zygotic mutant background. In the *chd7* mutant fish, we found shortened craniofacial cartilages and extra cartilage formation. Furthermore, the length of the ventral aorta is altered in *chd7* mutants. Many CHARGE patients have aortic arch anomalies. It should be noted that the aberrant branching of the first branchial arch artery is observed for the first time in *chd7* fish mutants. To understand the cellular mechanism of CHARGE syndrome, neural crest cells (NCCs), that contribute to craniofacial and cardiovascular tissues, are examined using *sox10:Cre* lineage tracing. In contrast to its function in cranial NCCs, we found that the cardiac NCC-derived mural cells along the ventral aorta and aortic arch arteries are not affected in *chd7* mutant fish. The *chd7* fish mutants we generated recapitulate some of the craniofacial and cardiovascular phenotypes found in CHARGE patients and can be used to further determine the roles of CHD7.

## 1 Introduction

CHARGE syndrome is an autosomal dominant genetic disease that is named after the abbreviation for six standard features of this disorder: coloboma, heart disease, choanal atresia, retarded growth, genital hypoplasia, and ear anomalies and/or deafness (Hsu et al., 2014). It is the leading syndrome associated with deafness–blindness in school-aged children in the United States (Flanagan et al., 2007). Babies born with CHARGE syndrome often have life-threatening defects and require intensive treatments including many surgeries. In addition to the six standard features, craniofacial defects including cleft palate/lip and asymmetric face palsy (Pagon et al., 1981) and cardiovascular malformations including outflow tract defects, atrioventricular septal defect, persistent ductus arteriosus (Corsten-Janssen et al., 2013), and aortic arch anomalies (Corsten-Janssen and Scambler, 2017) are frequently present in CHARGE patients. CHARGE syndrome is also extremely complex in terms of symptomatology, which can vary in severity from patient to patient, making it difficult to study.

Chromodomain helicase DNA-binding protein 7 (CHD7), a member of the SNF2-protein superfamily, is an ATP-dependent chromatin remodeler (Allen et al., 2007). *CHD7* has been found mutated in two-thirds of patients with CHARGE syndrome. A study involving 299 patients with pathogenic *CHD7* mutations shows that 74% of these patients have heart defects (Corsten-Janssen et al., 2013). Moreover, in addition to CHARGE syndrome patients, *CHD7* mutations have also been identified in non-syndromic patients with congenital heart defects (Zaidi et al., 2013; Yan et al., 2020).

To study the mechanisms underlying CHARGE syndrome *in vivo*, multiple *Chd7* N-ethyl-N-nitrosourea (ENU) mutants and *Chd7* null mouse models have been generated. While no homozygous embryos were detected beyond E10.5 (Hurd et al., 2007; Layman et al., 2010; Yan et al., 2020), the heterozygous *Chd7* mice recapitulate various symptoms of CHARGE syndrome, such as coloboma, inner ear abnormalities, cleft palate, choanal defects, cardiovascular defects, genital defects, and developmental growth delays (Bosman et al., 2005; Layman et al., 2009; Layman et al., 2010; Schulz et al., 2014b; Ogier et al., 2014; Gage et al., 2015). However, the postnatal lethality of *Chd7* mutant mice makes it difficult to study the overall function of *CHD*7 and screen the potential therapeutic compounds in mice.

Zebrafish have become an emerging model to study development and model human diseases. *In situ* hybridization experiments have shown that the *chd7* transcripts are broadly expressed in developing zebrafish, mainly in the central nervous system, gut, tail bud, and somite borders (Jacobs-McDaniels and Albertson, 2011). To study CHARGE syndrome, a few *chd7* zebrafish mutants have been generated and reported. In contrast to CHARGE patients with only one mutant allele of *CHD7*, *chd7* heterozygous mutant fish do not display obvious phenotypes, except for the anxiety-like and aggressive behavior (Liu and Liu, 2020). Nevertheless, *chd7* homozygous fish showed mild CHARGE-like phenotypes such as smaller eyes, enlarged heart with edema, failure to inflate the swim bladder (Prykhozhij et al., 2017), small head, cranial cartilage malformations, and cranial nerve defects (Jamadagni et al., 2021). In addition, decreased size of the gastrointestinal tract (Cloney et al., 2018), scattered *sox10*-positive cranial NCCs (Liu et al., 2019), and impaired T-cell and thymus development (Liu et al., 2018) were also reported in *chd7* homozygous fish mutants. However, no detailed craniofacial cartilage and cardiovascular phenotypes in *chd7* fish mutants have been reported so far.

It should be noted that the tissues affected in CHARGE syndrome, such as missing ear lobes, deafness, broad forehead, reduced dysmorphic jaw, and hypopigmentation in some patients, are mainly derived from the neural crest cell (NCC) lineage. NCC is a group of transient cells in vertebrates, which migrates out from the neural tube and then gives rise to diverse cell lineages and contributes to different tissues, including the craniofacial skeleton, cartilage, ear, cells in the great vessels, and the septum of the heart in humans (Huang and Saint-Jeannet, 2004; Achilleos and Trainor, 2012). Thus, NCCs may be one major cell type that contributes to concomitant craniofacial and congenital cardiac defects, which are present in patients with CHARGE and other genetic syndromes and can also occur in non-syndromic patients with cleft and heart defects (Munabi et al., 2022). Consistent with this notion, *in situ* hybridization data showed that *chd7* is expressed in rhombomeres, from which some NCCs are derived, at 24 days post fertilization (dpf) and in the developing pharynx at 48 to 96 hpf (Liu et al., 2018).

NCCs migrate to seven pharyngeal arches (PAs) in zebrafish. The first one contributes to the craniofacial cartilage including the Meckel’s cartilage and part of the palatoquadrate; the second PA forms the dorsal hyosymplectics, the ventral ceratohyals, and the interhyal cartilage; and the 3–7 PAs produce pairs of ventrolateral ceratobranchial cartilages. Specifically, the seventh PA contributes to the fifth ceratobranchial cartilage that forms several ossified pharyngeal teeth (Frisdal and Trainor, 2014; Mork and Crump, 2015). In mammals, PAs 3, 4, and 6 contribute to the formation of aortic arch arteries (AAs) and then remodel into the great vessels–the aortic arch, pulmonary arteries, and carotid arteries (Vincent and Buckingham, 2010). There are only five pairs of AAs in mammals since the sixth pair is never fully formed (Bamforth et al., 2013). However, in zebrafish, all six pairs of AAs that branch out from the ventral aorta (VA) are completely formed. The initial AA formation and patterning in zebrafish are very similar to those in mammals, but the later complex remodeling into the aorta and pulmonary artery does not happen; and AAs 3–6 carry blood to the gills instead (Anderson et al., 2008). Nonetheless, zebrafish is still an excellent model to study the initial formation of AAs and the great vessels (Nagelberg et al., 2015).

Here, we report the generation and characterization of new *chd7* mutants and the novel concomitant craniofacial and cardiovascular phenotypes. These defects are further enhanced in maternal zygotic *chd7* mutants. We discovered that *chd7* mutants have altered chondrocyte stacking, shortened craniofacial cartilages, and extra craniofacial cartilage. Furthermore, the length of the VA is decreased and the branching and patterning of AA3 (the first brachial arch artery) are impaired in *chd7* mutants. Interestingly, we found that NCC-derived mural cells (pericytes and smooth muscle cells) are not affected along the VA and AAs. Our *chd7* mutant provides the first model to mimic the concomitant craniofacial and cardiovascular phenotypes found in CHARGE and non-syndromic patients and can be used to further determine the underlying mechanisms of *CHD7*.

## 2 Materials and methods

### 2.1 Zebrafish maintenance

All procedures described have been approved by the CHLA IACUC committee. To raise zebrafish, we keep the fish at 28.5°C with a 14-h light/10 h dark cycle. Brine shrimp and commercial powder food smaller than 450 µm have been used to feed the fish. In our system, we feed the larvae with powder food smaller than 50 μm at the beginning and switch to powder food smaller than 100 µm together with brine shrimp for juvenile fish. Fish housing and maintenance are under standard care with CHLA IACUC oversight. After crossing the fish, fertilized embryos were collected and changed into the E3 media (5 mM NaCl, 0.17 mM KCl, 0.33 mM CaCl_2_, 0.33 mM MgSO_4_, and 10^–5^% methylene blue). An incubator at 28.5°C is used to raise the embryos, and plus and minus 5°C may be used to adjust the speed of the development.

Tricaine-s/syncaine (ms 222) fish anesthetic (Syndel) was used as anesthesia (0.168 mg/ml in fish water), and euthanasia (0.4 mg/ml in fish water for more than 15 min).

### 2.2 Fish strains

The AB line (ZIRC and UCLA) is used as the wild type (WT) control in this experiment. The following transgenic lines were used in this study: *Tg(kdrl:mTurquoise)* (Harrison et al., 2019), *Tg(pdgfrb:GFP)* (Ando et al., 2016), *Tg*(*Mmu.Sox10-Mmu.Fos:Cre*)*zf384* (Kague et al., 2012), and *Tg*(*-3.5ubb:loxP-STOP-loxP-mCherry*)*el818* (Fabian et al., 2020).

### 2.3 *chd7* mutant generation using CRISPR/Cas9 technology

The targeting sequences of CRISPR Cas9 gRNAs are shown in Table 1. Before injection, the gRNA was thawed, the mix was prepared with 1/10 EnGen Spy Cas9 Nucleases (Cas9 protein, BioLabs), 1/10 10X Buffer 3.1(NEB), 200–300 ng/μL for single gRNA injection or 125 ng/μL each for multiple gRNA injection, and RNase free ddH_2_O. The mix was placed at 37°C for 10 min. The gRNA was coinjected together with Cas9 protein into single-cell stage zebrafish embryos and 2 nL of injection mixture per embryo.

**TABLE 1:** CRISPR gRNA targeting sequencing on *chd7*.

gRNA	Targeting sequence	PAM area
gRNA-sr5-1	GAA​GAG​ACG​ATC​TAG​TCG​GC	AGG
gRNA-sr5-2	AGA​ATT​TCA​GAT​GAC​GAC​GA	TGG
gRNA-sr5-3	GAC​GAT​GGA​GAT​GAT​TCT​GC	AGG
gRNA-sr5-4	TCA​GAT​GTA​GTT​GGA​GAC​TT	TGG
gRNA-SR6-1	GGG​AGA​GGG​AGT​TCA​GGA​CA	TGG
gRNA-SR6-2	GGC​CAG​CCG​AAA​GAC​CAT​TC	AGG

gRNAs were injected together with Cas9 protein individually, and four most efficient gRNAs were then chosen to carry out co-injection to gain the highest knockout efficiency. To check the efficiency of creating mutations, the genomic DNA of the injected fish was collected at 2 days post fertilization (dpf). T7E1 assay was used to distinguish and cut at the mismatched DNA heteroduplexes (Vouillot et al., 2015). The WT band cannot be cut by T7 endonuclease I (BioLabs), while the mutant mismatched bands can be cut.

Founder fish were raised, genotyping was performed, and mutants were sequenced to confirm the mutation (Genewiz from Azenta).

### 2.4 qRT-PCR

Quantitative real-time reverse transcriptional PCR was carried out using 24 hpf fish and four dpf fish. The total mRNA was extracted using TRIzol (Invitrogen). An RNeasy kit (Qiagen) was used to purify the total mRNA. cDNA was generated using the SuperScriptTM III Reverse Transcriptase kit (Invitrogen), and then real-time PCR (RT-PCR) was performed using LightCycler 480 SYBR Green I master mix (Roche). Two sets of PCR primer combinations (*chd7-1* and *chd7-2* listed in Supplementary Table S1) were used to check *chd7* expression. Data were normalized to *ef1α* as the reference gene.

### 2.5 Confocal microscopy

Embryos from 18 hpf to 5 dpf were euthanized in 0.1% Tricaine S (Syndel) and then mounted in 1% agarose with tricaine in fish water. Confocal microscopes (Leica STELLARIS 5 inverted confocal microscope and Zeiss LSM 710 inverted confocal microscope) were used to image the embryos and larvae.

### 2.6 Alcian blue and alizarin red staining

The larvae were euthanized with tricaine and then fixed in 2% PFA at room temperature for 1 h. Then, the larvae were washed with 100 mM Tris (pH 7.5) and 10 mM MgCl_2_ for 10 min (min) and incubated overnight in Alcian stain (stock: 0.1 g Alcian blue 8GX, 2.6 ml H_2_O, bring the volume to 50 ml by using 95% EtOH; working solution: 10 ml Alcian blue stock, 32.6 ml 95% EtOH, 5 ml Tris-HCl, pH 7.5, 0.5 ml 1M MgCl_2_, 0.9 ml H_2_O) at room temperature. The fish were destained by 80% EtOH/100 mM Tris (pH 7.5)/10 mM MgCl_2_, 50% EtOH/100 mM Tris (pH 7.5)/10 mM MgCl_2_, and 25% EtOH/100 mM Tris (pH 7.5)/10 mM MgCl_2_ for 5 min each. The fish were moved into a well of 24-well plates and bleached by 3% H_2_O_2_/0.5% KOH under a light source until the eyes turn to light brown. The fish were washed twice with 1 ml 25% glycerol/0.1% KOH for 10 min each and nutated in alizarin stain (stock: 0.25 g alizarin red S to 50 ml H_2_O, working solution: 1 ml alizarin red stock, 12.5 ml glycerol, 5 ml Tris-HCl, pH 7.5, 31.5 ml H_2_O) at room temperature for 1 h. The fish were destained by washing with 1 ml 50% glycerol/0.1% KOH for 10 min twice. Then gradually move them into 100 % glycerol and image the fish in glycerol using a Zeiss Axioplan Upright Microscope in a bright field.

### 2.7 Quantification of chondrocyte stacking arrangement

To analyze the chondrocyte arrangement, the angle between the three neighboring chondrocytes was measured. Since the cells connecting to the basibranchials are round and not as organized as the chondrocytes in the other ceratobranchials, counting one-fourth to one-third of the chondrocytes on the basibranchial side was avoided. The angles were measured from the first more organized chondrocytes if possible. If organized chondrocytes still cannot be identified, one-third of the chondrocytes were skipped on the basibranchial side. The basibranchial side was treated as the base and the other end as the tip. 1. A line vertical to the cell membrane was drawn to connect the cell to the adjacent cell (choose the one near the base side if there is more than one cell). 2. Step 1 was repeated, and the angle between these two lines was measured. 3. Within the cells that are not measured, the one which is closest to the base was chosen and steps 1 and 2 were repeated until all the cells are measured or there are less than three cells in the tip.

### 2.8 Survival rate quantification

For *chd7* ^*+/sr5*^ incrossed fish, there are three offspring genotypes: WT, *chd7* ^*+/sr5*^, and *chd7* ^*sr5*^. Due to the difficulty in performing genotyping in young larvae, we separate the embryos into three groups randomly after the gastrula stage. Genotyping was then performed at 7 dpf, 14 dpf, and 30 dpf for each group. The entire larvae were lysed to extract genomic DNA at 7 dpf and 14 dpf, and the fin was cut and used to extract the genomic DNA at 30 dpf.

We also incrossed *chd7* ^*sr5*^ male and female fish to get the *MZchd7* ^*sr5*^ offspring, and WT and *chd7* ^*+/sr5*^ fish from separate crosses were used as non-sibling controls. The fish number was counted every 3 days to draw the survival rate from 0 dpf to 30 dpf.

### 2.9 Aortic arch patterning quantification

To determine the asymmetry of the AAs and the location of AA3 branching points, the length of the bifurcation point of AA3 from the intersection point of the opercular artery (ORA) to the VA along the VA was measured. The value of the length on the left side is plotted as X, and the one on the right side is plotted as Y. For some *chd7* ^*+/sr5*^ and *chd7* ^*sr5*^ fish with abnormal AA patterning, the AA3 branched out from the ORA instead of from the VA. Thus, the length was considered negative along the VA. Scatter plots were used to show the symmetry of AA patterning. The data point with negative X or Y values marks the fish with abnormal AA3 branching. All the data points outside of the gray (15%) error interval region are considered having asymmetric AAs.

### 2.10 Statistical analysis

Sample sizes, statistical tests, and *p*-values are stated in the figure legends. Data were measured using ImageJ (Schneider et al., 2012). One-way ANOVA followed by Dunnett’s multiple comparisons test and chi-squared test were performed using GraphPad Prism version 9.0.0 for Mac OS X, GraphPad Software, San Diego, California, United States , www.graphpad.comtest. Figures were drawn using Prism. Differences were considered statistically significant at *p* < 0.05. For the bar chart, the error bars are generated with the mean and standard error of the measurements. For the box and whisker plots, the whiskers are generated with the minimum to maximum value and all points were shown.

## 3 Results

### 3.1 *chd7* mutant fish show CHARGE like phenotypes

**FIGURE 1:**
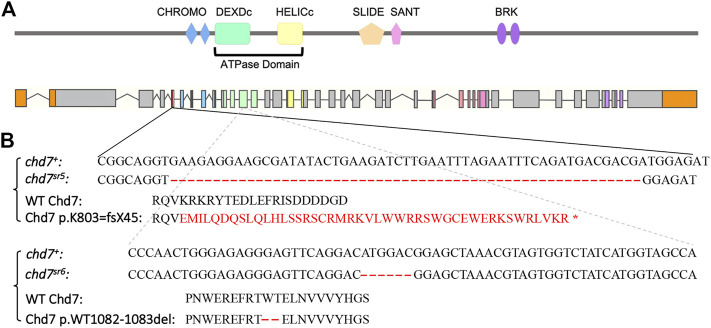
Generation of *chd7* zebrafish mutants. **(A)** Schematics of Chd7 protein and *chd7* gene structure. Chd7 protein contains double CHROMO domains, an ATPase domain including a DEXDc and HELICc domain, a SLIDE/SANT domain, and double BRK domains. **(B)** Sequence of the *chd7* ^*sr5*^ and *chd7* ^*sr6*^ mutants. *Chd7* ^*sr5*^ contains a 55-bp deletion in the fifth exon of *chd7*, which generated a truncation after the first chromo domain. The deletion created a 44-amino acid frame-shift followed by a stop codon. *Chd7* ^*sr6*^ has a mutation in the twelfth exon, which encodes the DEXDc domain, and causes a deletion of two amino acids, tryptophan and threonine.

The CRISPR/Cas9 system was used to generate *chd7* mutants by co-injecting Cas9 protein together with gRNAs into one-cell stage embryos. We modified a previously published method (Wu et al., 2018) by co-injecting two or four gRNAs targeting the same exon (Table 1) of the *chd7* gene to achieve high knockout efficiency. We identified two *chd7* mutant alleles; the first *chd7* ^*sr5*^ allele contains a 55-base pair deletion in exon 5 which encodes the first CHROMO domain of Chd7. This deletion is predicted to result in a frame-shift mutation that starts at the 803rd lysine and creates a stop codon after 45 amino acids (Figures 1A,B). Mutations in the ATPase domain might have a dominant negative effect by disrupting important protein–protein interactions (Bouazoune and Kingston, 2012). Thus, we generated the second *chd7* ^*sr6*^ allele carrying an in-frame deletion that removes two highly conserved amino acids (the 1082nd tryptophan and 1083rd threonine) in the ATPase domain of Chd7 (Figures 1A,B).

**FIGURE 2:**
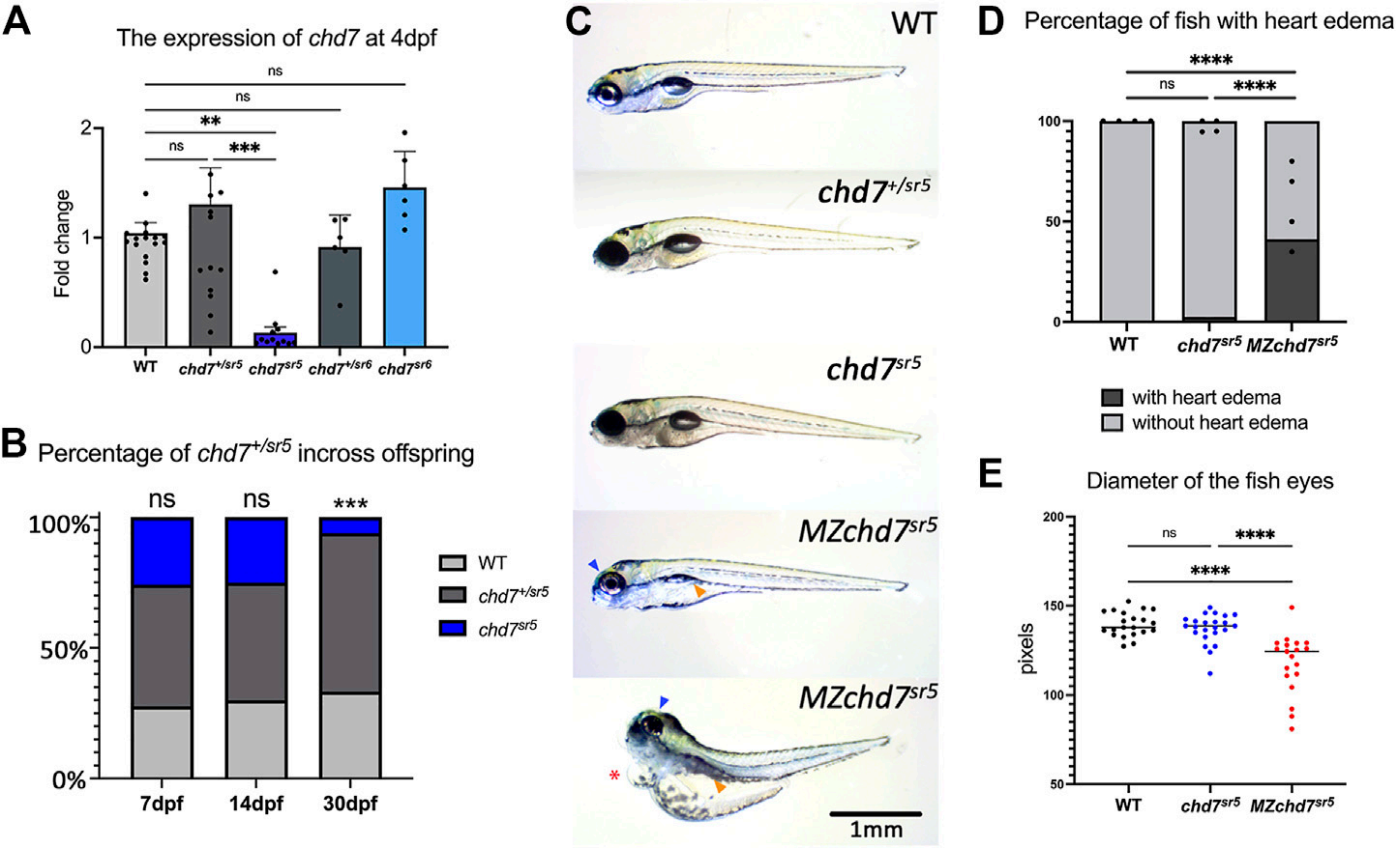
Overall phenotypes of *chd7* mutants. **(A)** Expression of *chd7* in *chd7* ^*sr5*^ and *chd7* ^*sr6*^ larvae at 4 dpf. The fold change of *chd7* expression compared to WT was shown. **(B)** Percentage of the surviving offspring from *chd7* ^*sr5*^incross (*n* > 40 for each time point). **(C)** Bright field images of *chd7* ^*sr5*^ and *MZchd7* ^*sr5*^ mutants at 7 dpf. *MZchd7* ^*sr5*^ fish have a smaller swim bladder (yellow arrowhead), pericardial edema (red asterisk), and small eyes (blue arrowhead). **(D)** Quantification of heart edema. Nearly 40% of *MZchd7* ^*sr5*^ fish have heart edema; however, *chd7* ^*sr5*^ fish do not show heart edema phenotypes. WT: *n* = 879, *chd7* ^*sr5*^: *n* = 747, *MZchd7* ^*sr5*^: *n* = 751. **(E)** Quantification of the small eye phenotype. The longest diameter of the dissected eye was measured. The eye size of the *MZchd7* ^*sr5*^ mutants is significantly reduced (*n* = 22 for each group). Statistical analyses were performed using the ordinary one-way ANOVA test for **(A,E)** and chi-square test for **(B,D)**. ns: *p* ≥ 0.05, *: *p* < 0.05, **: *p* < 0.01, ***: *p* < 0.001, and ****: *p* < 0.0001.

Quantitative reverse transcription PCR (qRT-PCR) of *chd7* in these two mutants at 4 dpf showed dramatically decreased *chd7* mRNA levels in *chd7* ^*sr5*^ homozygous mutants (*chd7* ^*sr5*^) but not in *chd7* ^*sr5*^ heterozygous (*chd7* ^*+/sr5*^) or *chd7* ^*sr6*^ homozygous (*chd7* ^*sr6*^) larvae (Figure 2A; Supplementary Tables S1, S2). This result suggests that *chd7* mRNA is degraded in the *chd7* ^*sr5*^ mutant. Upon further analyses, we observed significantly impaired survival of *chd7* ^*sr5*^ *.* We uncovered that 6% fish are surviving *chd7* ^*sr5*^ mutants compared to 25%, the predicted Mendelian ratio by 30 dpf (*p*-value <0.001) (Figure 2B). Despite the impaired survival, the surviving *chd* ^*sr5*^ fish are able to reproduce normally and display very mild gross phenotypes (Figure 2C). Since *chd7* transcripts can be detected in zygotes and 2-cell stage embryos (Rauch et al., 2003) and maternal *chd7* mRNAs could play important roles in early development processes, we examined whether *chd7* ^*sr5*^ might show maternal zygotic mutant phenotypes. We found that maternal zygotic *chd7* ^*sr5*^ (*MZchd7* ^*sr5*^) mutants show impaired survival similar to the zygotic *chd7* ^*sr5*^ mutant larvae (Figure 2B). The number of *MZchd7* ^*sr5*^ larvae dramatically decreased after 10 dpf (∼90% died), while only ∼15% of *chd*7^*+/sr5*^ and WT siblings died by 30 dpf (Supplementary Figure S1A). The *chd7* transcripts are also significantly decreased in *MZchd7* ^*sr5*^ mutants (Supplementary Figure S1B).

As we expected, the *MZchd7* ^*sr5*^ mutants showed more severe CHARGE-like phenotypes such as small head, small eyes, and cardiac edema (Figure 2C). This is consistent with other *chd7* fish mutants reported (Prykhozhij et al., 2017; Liu et al., 2018; Jamadagni et al., 2021). When comparing the pericardial edema in WT (*n* = 879), *chd7* ^*sr5*^ (*n* = 747), and *MZchd7* ^*sr5*^ (*n* = 751), the *MZchd7* ^*sr5*^ larvae have significantly more heart edema (∼40%) (Figure 2D). These data suggested that maternal *chd7* mRNA might function during the early development in *chd7* ^*sr5*^ larvae. This might also account for the mild phenotypes in previously published fish lines (Prykhozhij et al., 2017; Liu and Liu, 2020; Jamadagni et al., 2021). *MZchd7* ^*sr5*^ fish have smaller eyes compared to those of the WT (*n* = 22 for each group), but *chd7* ^*sr5*^ fish do not show this phenotype (*n* = 22) (Figure 2E). This small-eye phenotype was also observed in other *chd7* mutants (Prykhozhij et al., 2017). In summary, *chd7* ^*sr5*^ mutants showed mild gross CHARGE phenotypes, which can be enhanced in the *MZchd7* ^*sr5*^ mutants.

### 3.2 The *chd7* ^*sr5*^ mutants show CHARGE-like phenotypes in craniofacial structure

**FIGURE 3:**
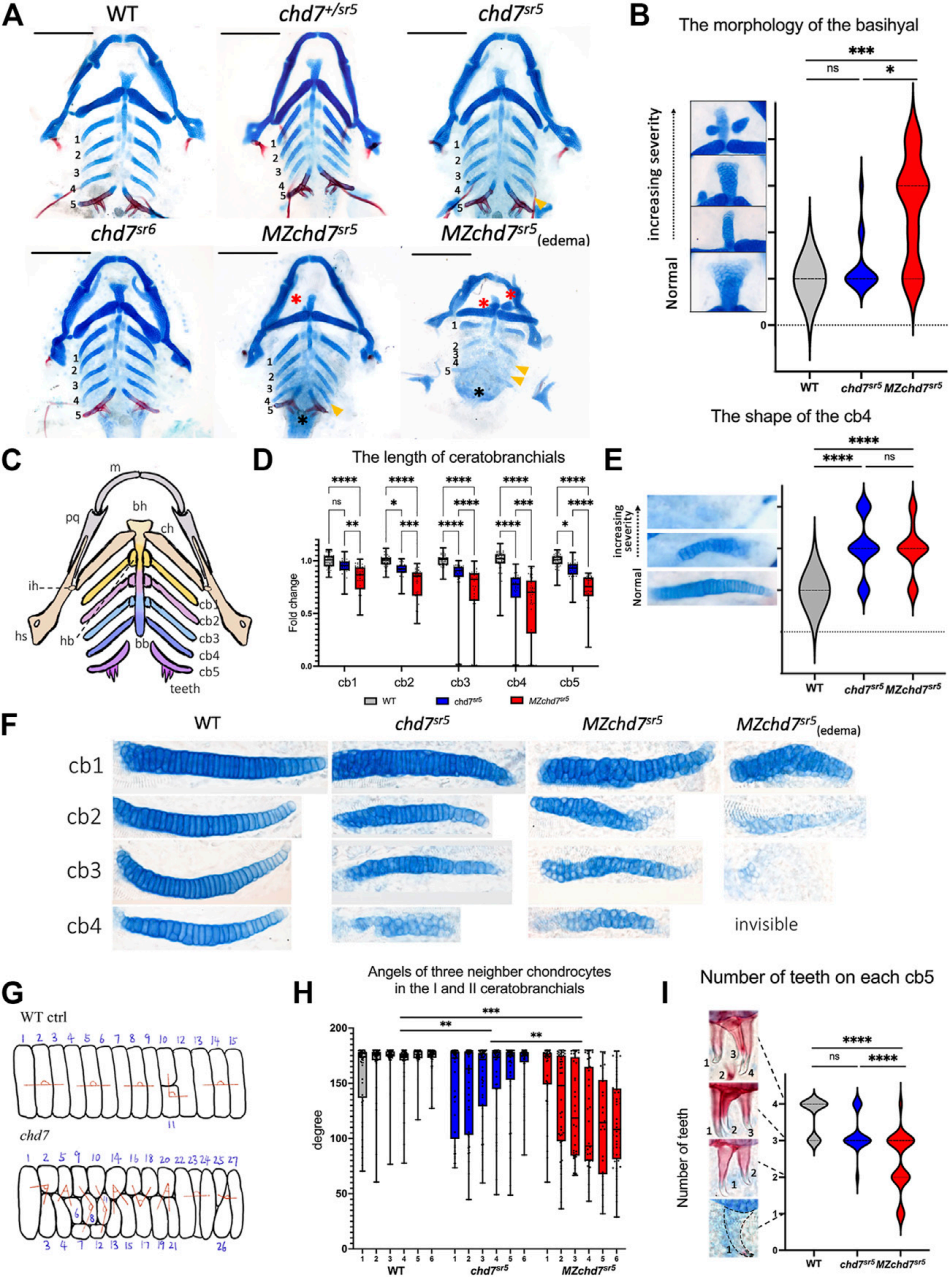
Craniofacial phenotype characterization in *chd7* ^*sr5*^ mutants. **(A)** Viscerocranium morphology. The viscerocranium was dissected out from the Alcian blue and Alizarin red costained WT, *chd7* ^sr5^, and *MZchd7* ^*sr5*^ mutants with or without heart edema at 7 dpf. Yellow arrowheads indicate the reduction in the IV ceratobranchial; black asterisks mark the defects in the teeth; red asterisks mark the abnormalities in the basihyal. **(B)** Quantification of the different types of the morphology of basihyal in WT, *chd7* ^sr5^, and *MZchd7* ^sr5^. (WT: *n* = 16; *chd7* ^*sr5*^: *n* = 16; *MZchd7* ^*sr5*^: *n* = 15). The statistical analysis was performed using the chi-square test. **(C)** Schematic of the viscerocranium cartilage at 7 dpf. bb, basibranchial; bh, basihyal; cb, ceratobranchial; ch, ceratohyal; hb, hypobranchial; hs, hyosymplectic; ih, interhyal; m, Meckel’s; and pq, palatoquadrate. **(D)** Quantification of the length of I to V ceratobranchials in WT, *chd7* ^sr5^, and *MZchd7* ^*sr5*^. (WT: *n* = 24; *chd7* ^*sr5*^: *n* = 20; *MZchd7* ^*sr5*^: *n* = 23). The statistical significance was performed using the ordinary one-way ANOVA test. **(E)** Quantification of different types of the morphology of the IV ceratobranchial. (*n* = 22 for each group). The statistical analysis was performed using the chi-square test. **(F)** High-magnification image shows that chondrocyte stacking is disrupted in *chd7* ^*sr5*^ mutants, and severely disorganized stacking has been found in *MZchd7* ^*sr5*^ mutants. **(G)** Schematic of chondrocytes’ stacking measurement. **(H)** Angles of three neighbor chondrocytes in I and II ceratobranchials. Each number indicates a different fish. (*n* = 6 for each group). The significance was calculated between different groups. *chd7* ^*sr5*^ mutants have mild disorganization in the chondrocyte arrangement, but *MZchd7* ^*sr5*^ mutants have more severe defects in the chondrocytes. The statistical analysis was performed using the ordinary one-way ANOVA test. **(I)** Alizarin red-stained teeth are reduced in *chd7* ^*sr5*^ mutants at 7 dpf. Nearly 50% of *MZchd7* ^*sr5*^ fish have defects in teeth number. (WT: *n* = 23; *chd7* ^*sr5*^: *n* = 20; *MZchd7* ^*sr5*^: *n* = 38). The statistical significance was performed using the chi-square test. ns: *p* ≥ 0.05, *: *p* < 0.05, **: *p* < 0.01, ***: *p* < 0.001, and ****: *p* < 0.0001.

Many patients with the CHARGE syndrome have a typical CHARGE face, which is asymmetric with a broad prominent forehead, a prominent nasal bridge with square root, a prominent nasal columella, flat midface, small mouth, and occasional small chin. In zebrafish, the neurocranium and viscerocranium together form the craniofacial cartilage. In *chd7* ^sr5^ and *MZchd7* ^*sr5*^ fish, the structure of neurocranium cartilage does not have dramatic defects compared to that of the WT. However, they have mild defects in the viscerocranium cartilage structure, especially in the basihyal, the ceratobranchials, and the teeth (Figures 3A–C) (Mork and Crump, 2015).

Using Alcian blue staining to visualize the cartilages, we found that the structure of the basihyal cartilage is impaired in *chd7* ^*sr5*^ and the defect is more severe in *MZchd7* ^*sr5*^ mutants. Based on their various severities, we categorized the morphology of the basihyal into four types (Figure 3B). Normal basihyal is triangle shaped; while in the fish with mild defects, the basihyal cartilage is narrowed. For larvae with moderate defects, an extra cartilage could also be found near the narrowed basihyal cartilage. For the larvae with the most severe defects, two extra cartilages were found on both sides of the narrowed basihyal. A few *chd7* ^*sr5*^ mutants have defects in the basihyal (3 in 16). However, this number is increased to around 2/3 in *MZchd7* ^*sr5*^ mutants (11 in 15). Moreover, more fish have moderate and severe phenotypes in *MZchd7* ^*sr5*^ (9 in 15) compared to *chd7* ^*sr5*^ mutants (1 in 16) (Figure 3B).

In addition to the basihyal phenotype, the quantification of the length of ceratobranchials I to V showed that both *chd7* ^*sr5*^ and *MZchd7* ^*sr5*^ mutants have shorter ceratobranchial cartilage (Figure 3D). Interestingly, the III and IV ceratobranchials are more severely decreased but not the V ceratobranchial, indicating that Chd7 may have more specific roles in the 3–6th PAs but not in the 7th PA. In addition to the shorter length, some of the IV ceratobranchials also have an abnormal shovel shape or are completely missing in both *chd7* ^*sr5*^ and *MZchd7* ^*sr5*^ mutants (Figure 3E). However, we cannot rule out the possibility that significantly shortened ceratobranchials in *MZchd7* ^*sr5*^ mutants is secondary to the heart edema phenotypes (Figure 3F).

We further investigated the cartilage defects in *chd7* ^*sr5*^ mutants by examining the chondrocyte arrangement. During maturation, the prechondrocytes go through a process called convergence-extension. Rather than *via* cell proliferation, an elongation of cartilages is driven from bulky precartilaginous condensations into stacks of disk-shaped chondrocytes (Mork and Crump, 2015). When checking the chondrocytes at higher magnification, we found that their stacking is disorganized in *chd7* ^sr5^ and *MZchd7* ^*sr5*^ mutants (Figure 3F). To quantify this disorganization, we measured the angles between three neighboring chondrocytes (Figure 3G) in ceratobranchials I and II using a method as described (Shull et al., 2022). The results indicated that *chd7* ^sr5^ mutants have abnormal chondrocyte stacking (Figure 3H) compared to the WT. Moreover, this phenotype is significantly enhanced in *MZchd7* ^*sr5*^ mutants.

CHARGE patients can have one or more congenital dental abnormalities (Inchingolo et al., 2014). Therefore, we checked if *chd7* ^*sr5*^ mutants have craniofacial phenotype in teeth. Like other vertebrates, the teeth of zebrafish arise through epithelial–mesenchymal interactions (Verstraeten et al., 2010). We analyzed the tooth number of the fifth ceratobranchial. The *chd7* ^*sr5*^ mutants do not show a significantly reduced number of teeth. However, *MZchd7* ^*sr5*^ mutants have a large variation in the number of teeth (Figure 3I). It should be noted that many of the *MZchd7* ^*sr5*^ fish with heart edema lost the alizarin red-stained bone tissue, and only the tip of the teeth are stained red (Figure 3I), and this is caused by developmental delay. Overall, *chd7* ^sr5^ mutants have abnormalities in chondrocytes and osteocytes. Moreover, some of these defects are more severe in *MZchd7* ^*sr5*^ mutants. These results indicated that the early development process affected by the maternal *chd7* mRNA may also contribute to the later craniofacial cartilage, eye, and tooth formation.

### 3.3 The *chd7* ^*sr5*^ mutant fish have defects in ventral aorta and aortic arch artery

**FIGURE 4:**
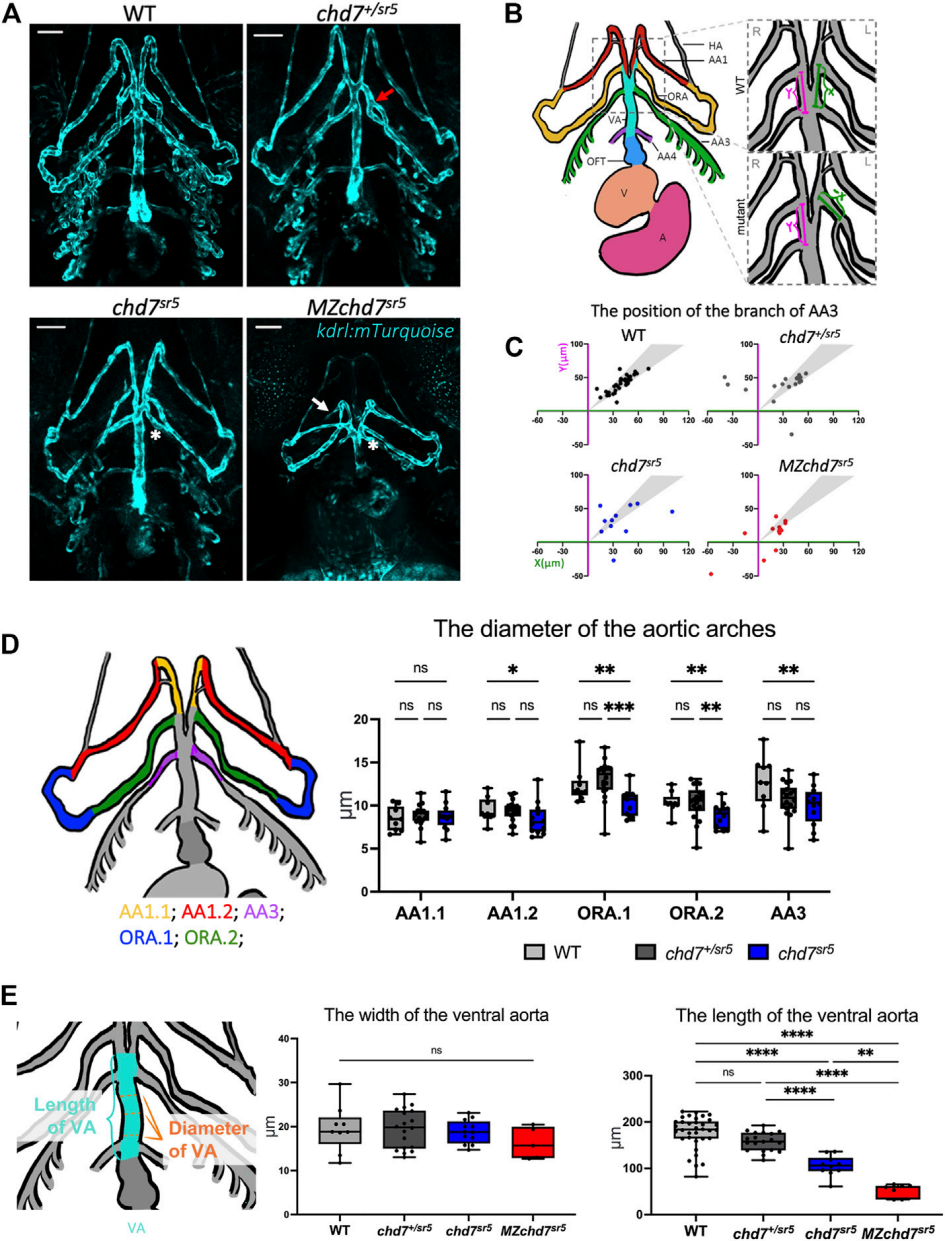
*chd7* ^*sr5*^ mutants show defects in arch artery patterning and ventral aorta length. **(A)** Ventral view of the *kdrl:mTurquois* fish at 102 hpf. 20x stacked confocal image, number of stacks: WT *n* = 25 (interval = 5 µm), *chd7* ^*sr5/+*^ *n* = 17 (interval = 7.5 µm), *chd7* ^*sr5*^ *n* = 20 (interval = 7.5 µm), and *MZchd7* ^*sr5*^ *n* = 21 (interval = 7.5 µm). Scale bar = 50 µm. **(B)** Schematic of ventral view of the zebrafish vasculature system at 102 hpf. The enlarged/zoomed in schematic illustrated the abnormal AA3 branching observed in some of the *chd7* mutants. The distance between the branching points of the ORA and AA3 along the VA was measured. *X* = the length on the left side of the fish, *Y* = the length on the right side of the fish. If the AA3 branches off from the ORA instead of the VA, a negative value is used to quantify the length. AA, aortic arch; AA1, mandibular arch; ORA, opercular artery; AA3, first branchial arch; AA4, second branchial arch; HA, hypobranchial artery; VA, ventral aorta; OFT, outflow tract; V, ventricle; and A, atrium. **(C)** Quantification of the branching positions of the AA3 on the left and right side of WT, *chd7* ^sr5^, and *MZchd7* ^*sr5*^ as *X* and *Y* coordinates. Each spot in the scatter diagram indicates one single fish. The gray area represents the range of symmetric distributions, in which the differences of the distance between *X* and *Y* is less than 15%. (WT: *n* = 34, *chd*7^*+/sr5*^: *n* = 19*, chd7* ^*sr5*^: *n* = 11*,* and *MZchd7* ^*sr5*^: *n* = 14). **(D)** Schematic and data quantification of the diameter of the AA1, ORA, and AA3. Due to the variation in the diameter at different segments of the vessels, AA1 and ORA were separated into AA1.1 and AA1.2 and ORA.1 and ORA.2, respectively. (WT: *n* = 9, *chd*7^*+/sr5*^: *n* = 18*,* and *chd7* ^*sr5*^: *n* = 12). The statistical analysis was performed using the two-way ANOVA test (mixed-effects analysis). **(E)** Schematic and data quantification of the width and length of the OFT. The mean diameter of the VA was used for quantification. The statistical analysis was performed using the ordinary one-way ANOVA test. (WT: *n* = 34, *chd*7^*+/sr5*^: *n* = 19*, chd7* ^*sr5*^: *n* = 11*,* and *MZchd7* ^*sr5*^: *n* = 14). ns: *p* ≥ 0.05, *: *p* < 0.05, **: *p* < 0.01, ***: *p* < 0.001, ****: *p* < 0.0001.

Some of the patients with *CHD7* mutations have defects in the outflow tract (OFT) and aortic arch artery (AA) (Corsten-Janssen et al., 2013). For instance, the aberrant subclavian artery has been observed in around 8% of CHARGE patients with congenital heart defects (Corsten-Janssen et al., 2013). Similarly, *Chd7* knockout mice have been shown to display abnormal AA development and AA4 is hypoplastic (Randall et al., 2009). To observe AA morphology, we utilized a *kdrl:mTurquoise* reporter line (Harrison et al., 2019) to label the endothelial cells of the vessel (Figure 4A). In zebrafish, the OFT is mainly composed of the bulbus arteriosus that connects the heart ventricle and the ventral aorta (VA) (Figure 4B). At 102 hpf, several AAs can be observed with the *kdrl:mTurquoise* reporter, which are the mandibular aortic arch (AA1), opercular artery (ORA), and branchial aortic arch arteries (AA3 and AA4) (Figures 4A,B).

In *chd7* ^*sr5*^ fish, we found that the patterning of ORA and AA3 is abnormal. In WT (*n* = 34), all AA3 branched out from the VA; however, in some *chd7* ^*+/sr5*^ larvae, AA3 branched out from the ORA instead, and this may affect the blood circulation (Figure 4A). Moreover, the AA3 branching patterns are asymmetric in *chd7* ^*+/sr5*^ and *chd7* ^*sr5*^ mutants (Figure 4A). To quantify the asymmetric patterning and branching defects, the length of the branching point of the AA3 from the intersection point of ORA and VA along the VA was measured for both left and right sides of the fish. Compared to the WT, *chd7* ^*+/sr5*^ *, chd7* ^*sr5*^ *,* and *MZchd7* ^*sr5*^ fish have more asymmetric branching points, and some also have abnormal AA3 branching (Figure 4C). In *chd7* ^*+/sr5*^, four out of 19 fish have abnormal AA3 branching. Furthermore, in *MZchd7* ^*sr5*^, nearly one-third have AA3 bifurcated from the ORA (four out of 14) (Figure 4C). These data indicated that the AA patterning is affected even in the *chd7* ^*+/sr5*^ fish and blood circulation to the craniofacial region and gill could be altered due to this abnormality.

To check whether other AAs are also affected in our *chd7* mutants, the diameter of the AAs was measured along different segments of the vessels (Figure 4D). Except for the first segment of the AA1 (AA1.1), we found that all other segments of the AA1 (AA1.2), ORA, and AA3 are thinner in *chd7* ^*sr5*^ fish but not in *chd7* ^*+/sr5*^ fish. Since the VA connects the OFT and AAs, we also measured the width and length of the VA (Figure 4E). The width of the VA does not show a significant difference between the WT and *chd* ^*sr5*^ mutants*.* In contrast, the length of the VA is significantly reduced in *chd7* ^*sr5*^ mutants and is even more decreased in *MZchd7* ^*sr5*^ fish (Figure 4E). In summary, AA patterning is impaired and VA length is decreased in *chd7* ^*sr5*^ mutants, and all these abnormalities together could influence blood circulation in *chd7* ^*sr5*^ mutants.

### 3.4 The cardiac neural crest cells and mural cells are not affected in *chd7* ^*sr5*^ ventral aorta

*CHD7* has been shown to have cell-autonomous functions in human embryonic stem cell-derived NCCs *in vitro* (Bajpai et al., 2010)*.* However, its roles in cardiac NCCs are controversial. A previous report showed that two waves of cardiac NCC migrations are observed in zebrafish, and the second wave of cardiac NCCs contributes to the smooth muscle cells of the VA (Cavanaugh et al., 2015). To determine whether cardiac NCC-derived cells are affected in *chd7* ^*sr5*^ mutants, we used *Tg* [(*Mmu.Sox10-Mmu.Fos:Cre*) *zf384*, *-3.5ubb:loxP-STOP-loxP-mCherry*) *el818*] (*sox10:switch* for short) to label and follow these cells. Furthermore, a *pdgfrb:GFP* reporter line was also used to label mural cells that include vascular smooth muscle cells and pericytes covering the endothelial cells and regulate vascular stability and homeostasis (Ando et al., 2016; Kapuria et al., 2022).

**FIGURE 5:**
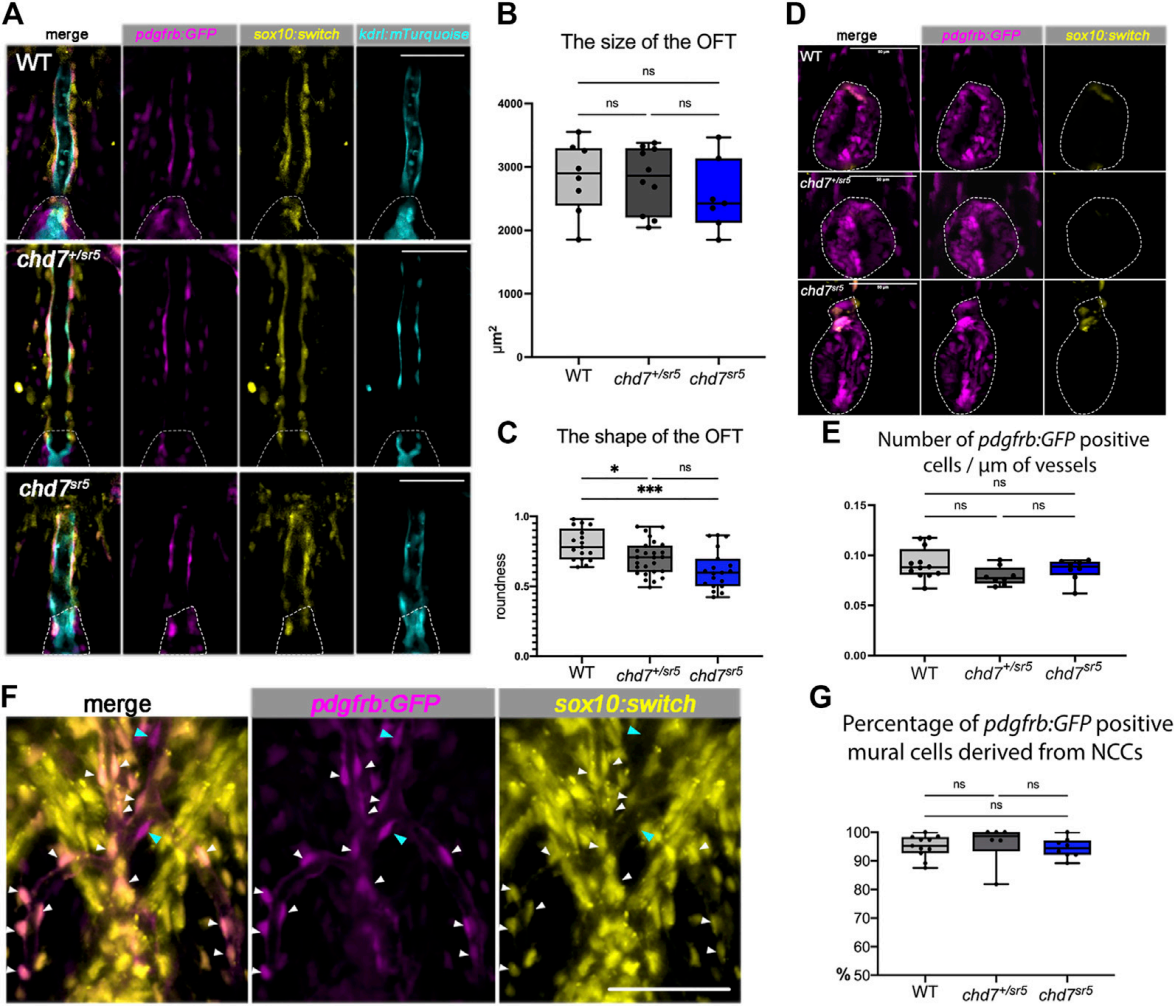
Cardiac NCCs and mural cells are not affected in the VA and OFT of *chd7* ^*sr5*^ mutants at 7 dpf. **(A)** *sox10:switch* (pseudo-colored yellow) positive NCCs contribute to *pdgfrb:GFP* (pseudo-colored magenta) positive mural cells around the VA in WT*, chd7* ^*+/sr5*^, and *chd7* ^*sr5*^ fish. 40X single-slice confocal images at the thickest z position of the VA. Dotted lines circled the OFT. **(B)** Quantification of the size of the OFT in *WT, chd7* ^*+/sr5*^, and *chd7* ^*sr5*^ fish. (WT: *n* = 8, *chd7* ^*+/sr5*^: *n* = 10, and *chd7* ^*sr5*^: *n* = 7). **(C)** Roundness analysis of the shape of OFT in WT*, chd7* ^*+/sr5*^, and *chd7* ^*sr5*^ fish. (WT: *n* = 17, *chd7* ^*+/sr5*^: *n* = 26, *chd7* ^*sr5*^: *n* = 19). **(D)** Very few *sox10:switch-*labeled NCCs contribute to the OFT (circled by the dotted lines). 40X single-slide confocal images at the widest z position of the OFT. **(E)** Quantification of the distribution of the *pdgfrb:GFP-*positive cells along the vessel. The distribution is determined using the total length of the vessel divided by the total *pdgfrb:GFP-*positive cells. (WT: *n* = 12, *chd7* ^*+/sr5*^: *n* = 8; *chd7* ^*sr5*^: *n* = 8). **(F)** Representative images of the *pdgfrb:GFP* and *sox10:switch-*positive cells along the aortic arches. White arrowheads indicate NCC-derived (*sox10:switch*) *pdgfrb:GFP-*labeled mural cells; blue arrowheads indicate the *pdgfrb:GFP* single positive mural cells. 40X confocal stacked images, stack number *n* = 2, interval = 6 µm. **(G)** Quantification of the percentage of *pdgfrb: GFP* positive mural cells derived from NCCs (*sox10: switch* positive) along the AAs. Scale bar = 50 µm for all images. The statistical analysis was performed using the ordinary one-way ANOVA test for all data quantifications. (WT: *n* = 11, *chd7* ^*+/sr5*^: *n* = 6; *chd7* ^*sr5*^: *n* = 8). ns: *p* ≥ 0.05, *: *p* < 0.05, **: *p* < 0.01, ***: *p* < 0.001, and ****: *p* < 0.0001.

As expected, *sox10:switch* labeled the cells around the VA, partially overlapping with the *pdgfrb:GFP* positive mural cells but not with the *kdrl:mTurquoise* positive endothelial cells (Figure 5A). Interestingly, the distribution of these *sox10:switch* positive cells is neither reduced nor increased in *chd7* ^*sr5*^ (Figure 5A) and *MZchd7* ^*sr5*^ (Supplementary Figure S2) mutants. On the other hand, although the size of the OFT remains the same in both WT and *chd7* ^*sr5*^ mutants (Figure 5B), the shape of the OFT changes significantly in *chd7* ^*+/sr5*^ and *chd7* ^*sr5*^ fish. The OFT is narrowed and elongated in the mutants (Figures 5C,D). Similar to the previous report (Cavanaugh et al., 2015), very few *sox10:switch* cells contribute to the smooth muscle cells in the OFT in zebrafish (Figure 5D). Therefore, the abnormally elongated shape of the OFT in *chd7* ^*+/sr5*^ and *chd7* ^*sr5*^ fish is not a result of changes in NCC-derived mural cells.

Although AA patterning is affected in *chd7* ^*sr5*^ fish, the distribution of the *pdgfrb: GFP-*positive cells on AAs is not affected (Figure 5E). Consistent with previously published data (Ando et al., 2016), we also observed that the NCC lineage traced (*sox10:switch* positive) cells contributing to the *pdgfrb: GFP-*labeled mural cells on AAs. Around 95% of *pdgfrb:GFP* positive cells are NCC-derived (*sox10:switch* positive) (Figure 5F), and the percentage is not affected in *chd7* ^*sr5*^ mutants (Figure 5G). These data together suggested that the cardiac NCCs and the NCC-derived mural cells are not affected in *chd7* ^*sr5*^ mutants. Thus, the abnormal AA branching may not be caused by the defects in NCC-derived mural cells.

### 3.5 *chd7* ^*sr5*^ mutants have extra craniofacial cartilage

**FIGURE 6:**
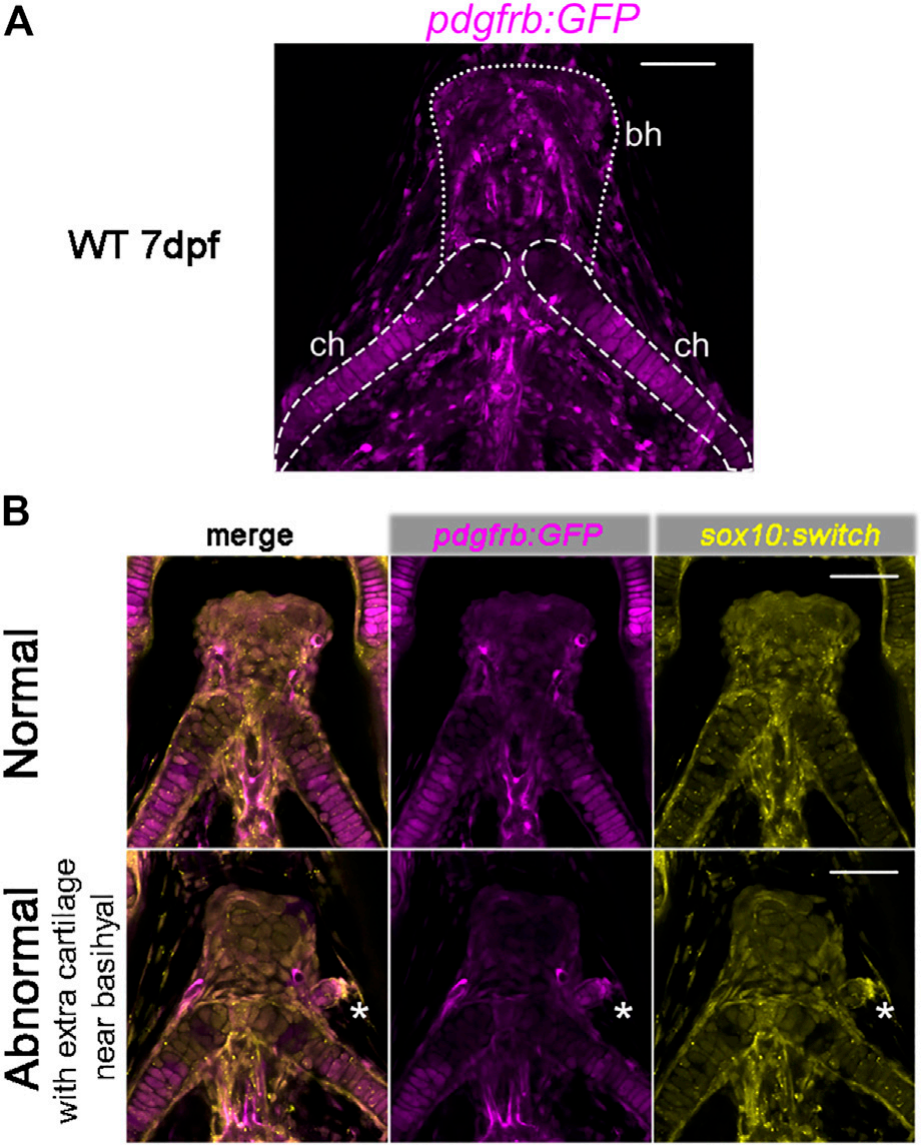
Expression of *pdgfrb:GFP* in craniofacial cartilages. **(A)** *pdgfrb:GFP* (pseudo-colored magenta) expression level is higher in the posterior part of the ceratohyal. 20X confocal stacked images, number of stacks n = 7, interval = 12 µm. **(B)** Representative images for the normal and abnormal basihyal in *chd7* ^*sr5*^ mutant fish. The abnormal one has extra cartilage near the basihyal cartilage, which expresses more *pdgfrb:GFP* compared to the basihyal cartilage and the nearby ceratohyal. *sox10:switch* (pseudo-colored yellow) labels NCCs. The white asterisks mark the extra cartilage. 20X single-slice confocal image. ch: ceratohyal; bh: basihyal (the number of fish with extra cartilage: WT: 0 in 6, *chd7* ^*+/sr5*^: 1 in 13, and *chd7* ^*sr5*^: 4 in 14). Scale bar = 50 µm for all images.

Previously, *pdgfrb* has been shown to be expressed in cranial NCCs within the PAs in zebrafish and the perichondrial layer surrounding the hyoid bone and nasal cartilage in E13.5 and E14.5 mice (McCarthy et al., 2016). Using the *pdgfrb:GFP* reporter fish, we found that the *pdgfrb* expression level is higher in the posterior part of the ceratohyal cartilage and the peripheral basihyal, but not in the anterior part of the ceratohyal and the central part of the basihyal (Figure 6A).

Intriguingly, in some of the *chd7* ^*sr5*^and *MZchd7* ^*sr5*^mutant fish, one or two extra isolated cartilages have been found next to the basihyal cartilage. These extra cartilages express more *pdgfrb:GFP* than the basihyal and the nearby anterior ceratohyal (Figure 6B). This enhanced *pdgfrb* expression may reflect a homeotic transformation of these cartilages from the posterior side into a more anterior position.

## 4 Discussion

CHARGE syndrome is a complex genetic disease. It is not only the leading cause of deafness–blindness disease in school-age children in the US but is also a disorder of the craniofacial structure and congenital heart problem that could be life-threatening and needs treatments immediately after birth. *CHD7* is the gene mutated in two-thirds of CHARGE patients. We have generated new *chd7* mutants and characterized novel concomitant craniofacial and cardiovascular phenotypes. We found that these defects are further enhanced in maternal zygotic *chd7* mutants. Although 75%–80% of the patients with CHARGE syndrome have congenital heart diseases, the heart defects are not extensively studied in previous zebrafish CHARGE models. We observed aberrant branching of the first branchial arch in both *chd7* heterozygous and homozygous fish mutants for the first time. Moreover, our zebrafish CHARGE model can be used to investigate the pathogenesis and identify potential therapeutic targets in the future.

### 4.1 Genetic compensation may cause weaker CHARGE-like phenotype

Morpholino (MO) was previously used to block *chd7* expression in zebrafish. *chd7*-MO injected at a higher dose (5–10 ng) induced lethality within 24 hpf, and embryos injected at a lower dose showed phenotypes such as eye abnormalities, otolith abnormalities, craniofacial defects (Patten et al., 2012; Asad et al., 2016), heart defects, and pectoral fin hypoplasia (Balasubramanian et al., 2014). None of these phenotypes can be rescued by coinjecting *chd7* and *p53* morpholinos (Patten et al., 2012; Balow et al., 2013), indicating that these phenotypes are not caused by random cell apoptosis. Due to concerns of off-target effects causing more severe phenotypes in the morphants, several *chd7* mutants were generated by different groups. However, none of the mutants has shown similar severity of CHARGE-like symptoms comparable to that of the morphants.

Genetic compensation triggered by nonsense-mediated decay of mutant mRNA has been reported to occur in zebrafish CRISPR mutants and might result in no or weak phenotypes (El-Brolosy et al., 2019). The previously published three *chd7* mutants and the *chd7* ^*sr5*^ described here all cause frameshift mutations. RT-PCR data showed that the *chd7* transcripts in the *chd7* ^*sr5*^ allele are degraded. Thus, genetic compensation might be triggered in *chd7* ^*sr5*^ mutants, and this might result in the weak CHARGE-like phenotypes we observed. The *chd7* transcripts in the *chd7* ^*sr6*^ allele are not degraded and thus should not trigger the genetic compensation. Therefore, *chd7* ^*sr6*^ mutants could, in theory, display more severe phenotypes. Yet, the craniofacial cartilage phenotypes in *chd7* ^*sr6*^ mutants are also mild. It is unknown whether the in-frame deletion in *chd7* ^*sr6*^ mutants does not abolish the Chd7 function completely. Nonetheless, CHARGE patients display large variations in symptoms, which may be due to different *CHD7* mutations. Analyzing patient-specific mutants mimicking patients’ alleles in the future will provide more insight into the pathogenesis.

### 4.2 Potential candidate genes that can compensate for loss of *chd7*

CHARGE syndrome has been shown to share some phenotypes overlapping with other congenital diseases such as Kabuki, DiGeorge, Alagille, Pallister–Hall, Feingold syndromes (Lettieri et al., 2021), and Mowat–Wilson syndromes. *KMT2D*, the gene mutated in Kabuki syndrome, has been suggested to function in the same chromatin modification machinery (Schulz et al., 2014a). Furthermore, it was reported that *CHD7* ^*LOF*^ and *KMT2D* ^*LOF*^ DNA methylation signatures share common CpG targets, specifically within *HOXA5* and *SLITRK5* (Butcher et al., 2017), providing more evidence that *CHD7* and *KMT2D* could have overlapping functions and may compensate each other. *ZEB2*, the gene mutated in Mowat–Wilson syndromes (Birkhoff et al., 2021), may compensate for *CHD7*. The *zeb2* is one of the downstream target genes of *chd7* (He et al., 2016) in zebrafish. Moreover, *ZEB2* regulates NCC formation and can bind to SMAD proteins (Birkhoff et al., 2021) as *CHD7* (Lettieri et al., 2021). These data suggest that *zeb2* could be another candidate gene to compensate for the loss of *chd7. SOX2*, which is mutated in Alagille, Pallister–Hall, and Feingold syndromes, regulates similar genes that are misexpressed in neural stem cells after shRNA-mediated knockdown with *Chd7* (Lettieri et al., 2021)*.* Although *CHD7* displays broader genomic binding sites than *SOX2*, *SOX2* could be a possible candidate gene to partially compensate for the defects caused by *CHD7* depletion.

Other candidates are the other members in the *CHD* family. In *Drosophila, kismet* is the only gene related to the subgroup III CHD members, and its function is overtaken in mammals by *CHD6–CHD9* (Batsukh et al., 2010)*.* The previous publication showed that *CHD7* interacts with *CHD8* to build a core component of a complex of similar function to *kismet* (Batsukh et al., 2010)*.* Similar combinations of two members from the same *CHD* subgroup to form a complex have also been observed between *CHD3* and *CHD4* (Hall and Georgel, 2007). Therefore, we could not rule out the possibility that other members from the subgroup III CHD family (*CHD6*, *CHD8*, and *CHD9*) could bind to each other and compensate for *CHD7*’s function.

### 4.3 Chd7 does not show cell-autonomous roles in cardiac NCC

Chd7 was previously shown as required in the pharyngeal ectoderm for normal AA development (Randall et al., 2009). Remarkably, the abnormal AA development in *Chd7* knockout mice cannot be rescued by NCC-specific expression of *Chd7* (Randall et al., 2009). Moreover, in a conditional *Chd7* knockout mouse model using an improved *Wnt1:Cre* (Lewis et al., 2013), the conotruncal defects are found to be caused by a dramatically reduced NCC migration into the proximal OFT cushions at E11.5 but not by impaired migration of NCCs into AAs (Yan et al., 2020). These results raised the question about the cell-autonomous roles of *C*hd7 in cardiac NCCs in different structures.

In mammals, a subpopulation of NCC continues to migrate to the OFT of the heart (Vincent and Buckingham, 2010). In zebrafish, two waves of cardiac NCC migrations were observed, and the second wave of cardiac NCCs contribute to the VA (Cavanaugh et al., 2015). Unlike in mice and humans, we found that very few cardiac NCCs contribute to the OFT in zebrafish. Although the data using *Chd7* conditional knockout mice suggest a cell-autonomous function of Chd7 in cardiac NCC (Yan et al., 2020), the proximal region of OFT, the conus arteriosus, is thought to be lost or form a segment distinct from the bulbus during the evolution in fish (Icardo, 2006), and the septation of the heart also does not occur (Grant et al., 2017). Interestingly, cardiac NCCs have been found to contribute to cardiomyocytes in the ventricle (Cavanaugh et al., 2015; Tang et al., 2019). This anatomical difference might explain the cardiac NCC contribution in fish and mammals. Furthermore, we found that the NCC-derived mural cells in both the VA and AAs are not affected in *chd7* ^*sr5*^ zebrafish. On the other hand, craniofacial NCCs have been shown as defective in both *chd7* morphant fish (Asad et al., 2016) and human cells *in vitro* (Bajpai et al., 2010). Thus, *CHD7* could have different cell-autonomous or non-cell-autonomous roles in cranial and cardiac NCCs.

## 5 Conclusion and significance

In summary, we generated two *chd7* mutant fish lines as the CHARGE syndrome disease model. Here, we describe the co-occurrence of craniofacial abnormalities and heart defects in new zebrafish *chd7* mutants. We described the possible maternal zygotic function of *chd7* for the first time. Furthermore, we investigated the cardiovascular phenotypes in detail and observed aberrant branching of the first branchial arch for the first time in *chd7* fish mutants. Many CHARGE patients have aortic arch artery anomalies. Intriguingly, we found extra cartilage formation in *chd7* mutants, which may be a homeotic transformation and worth further investigation for craniofacial phenotypes. Our zebrafish CHARGE model can be used to investigate the pathogenesis and identify potential therapeutic targets in the future.

## Data Availability

The raw data supporting the conclusion of this article will be made available by the authors, without undue reservation.
