# Supplementary material for: Craniofacial and cardiac defects in chd7 zebrafish mutants mimic CHARGE syndrome
Source: Front Cell Dev Biol. 2022 Dec 7;10:1030587. doi: 10.3389/fcell.2022.1030587 (PMC9768498; doi:10.3389/fcell.2022.1030587)
Supplement: Supplementary file 1 [file DataSheet1.pdf]

frontiers

Supplementary Material

1 Supplementary Figures and Tables

1.1 Supplementary Figures

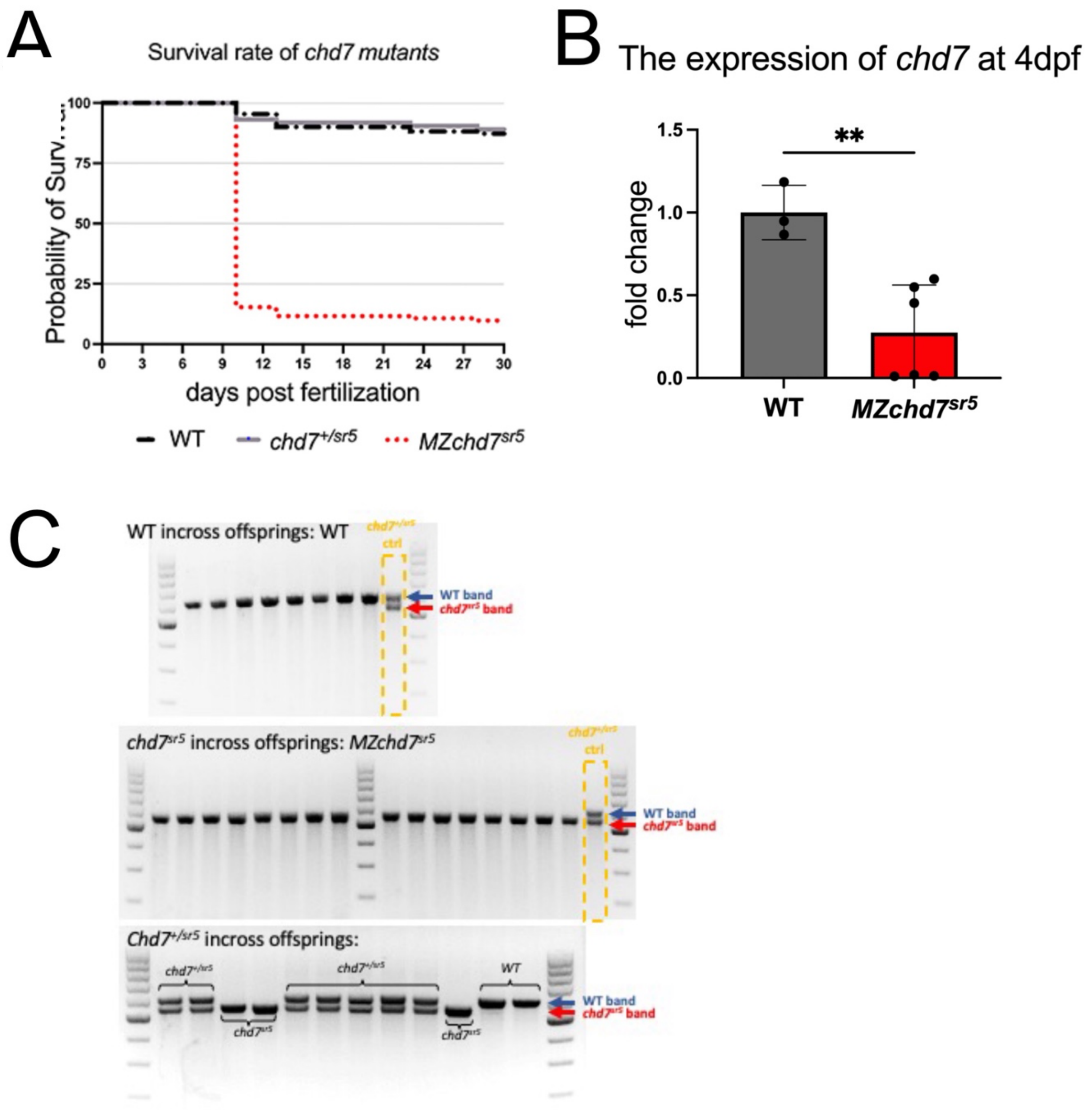

Supplementary Figure 1. Survival rates analysis of non-sibling WT, *chd7*$^{+/sr5}$, and *MZchd7*^{sr5} fish. (n=37 for each group, triplicates were quantified). The *MZchd7*^{sr5} fish showed similar mortality as the *chd7*^{sr5} fish in Figure 2. **B.** The expression of *chd7* in WT and *MZchd7*^{sr5} fish at 4 dpf. **C.** Genotyping of the WT incross, *chd7*$^{+/sr5}$ incross, and *chd7*^{sr5} incross offspring.

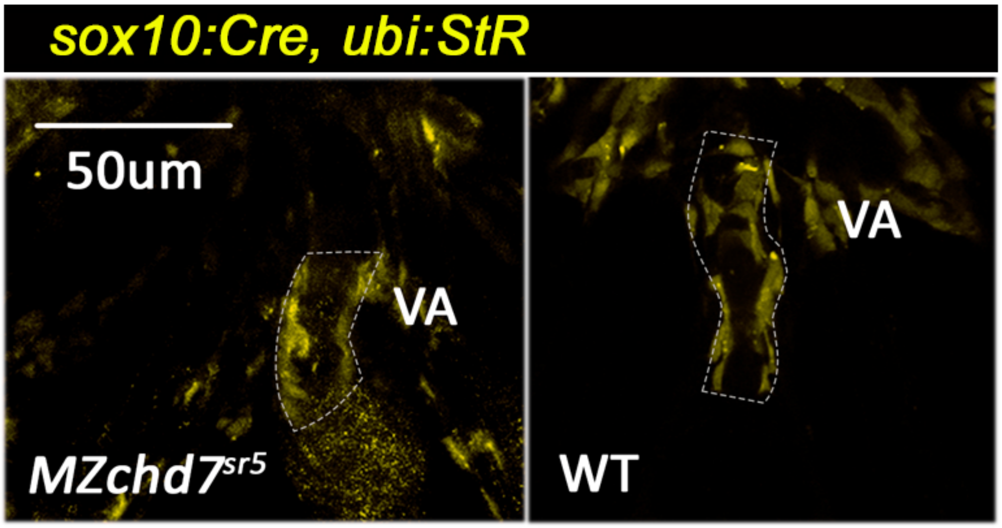

Supplementary Figure 2. The *sox10:Cre* lineage traced cells along the VA in WT and *MZchd7*^{sr5} at 4 dpf. Dotted line circled the VA. 40X Z stacked confocal image. Stack number n=4.

1.2. Supplementary Tables

Supplementary Table 1: Primers used in the qRT-PCR experiment.

**Primer**	**sequence**
ef1α Forward primer	AGCTGATCGTTGGAGTCAAC
ef1α Reverse primer	TGCGCTGACTTCCTTGGTG
chd7 Forward primer 1	ACCGTGCCATCTTGGAGAAG
chd7 Reverse primer 1	TCATGGCCTGGAGGTGAAAC
chd7 Forward primer 2	AGTGCTGCAATCACCCCTAC
chd7 Reverse primer 2	CAAAGCGGTCAGAGTCTGGT

Supplementary Table 2: qRT-PCR Raw data: Ct value. The replicate Ct values are shown in each cell.

**Genotype**	***ef1α***			***chd7-1***			***chd7-2***		
**experiments**	**No.1**	**No.2**	**No.3**	**No.1**	**No.2**	**No.3**	**No.1**	**No.2**	**No.3**
**WT**	**16.16<br>17.21<br>17.22**	**16.6<br>17.29<br>17.19**	**16.54<br>16.48<br>16.45**	**26.46<br>28.43<br>28.31**	**24.17<br>24.44<br>24.43**	**23.52<br>23.46<br>23.57**	**26.09<br>25.96**	**23.97<br>24<br>23.99**	**24.66<br>25.42<br>25.52**
$chd7^{+/sr5}$	**17.68<br>21.53<br>19.54**	**17.63<br>16.64<br>17.71<br>16.85**		**28.19<br>31.55<br>30.15**	**24.28<br>23.87<br>25.04<br>24.03**		**27.11<br>31.62<br>29.69**	**24.63<br>23.7<br>25.97<br>24.67**	
$chd7^{sr5}$	**18.25<br>19.09<br>19.03**	**16.9<br>17.45<br>17.44**		**30.2<br>32.53<br>32.28**	**28.94<br>29.56<br>29.57**		**30.2<br>32.76<br>31.79**	**28.08<br>28.47<br>28.9**	
$chd7^{+/sr6}$			**15.72<br>15.67<br>15.7**			**22.91<br>22.72<br>22.86**			**25.75<br>24.13<br>24.14**
$chd7^{sr6}$			**15.61<br>15.63<br>15.63**			**22.55<br>22.23<br>22.09**			**24.01<br>23.51<br>23.31**
